# The effect of experimental conditions on the formation of dixanthogen by triiodide oxidation in the determination of ethyl xanthate by HPLC–ICP-MS/MS

**DOI:** 10.1007/s44211-022-00155-x

**Published:** 2022-07-07

**Authors:** Ronja Suvela, Simon Genevrais, Matti Niemelä, Paavo Perämäki

**Affiliations:** 1 Research Unit of Sustainable Chemistry, University of Oulu, P.O Box 3000, 90014 Oulu, Finland; 2 University of Poitiers, Poitiers, France

**Keywords:** Xanthate, Triiodide oxidation, HPLC–ICP-MS/MS, Sulfur

## Abstract

The rising concern over the environmental impact of xanthates, especially in the arctic region, has increased the need to study these traditional flotation reagents in greater detail. The environmental concern relates mostly to the formation of carbon disulfide (CS_2_) and the heavy metal complexes of xanthates. Due to the unstable nature and multiple reaction mechanisms of xanthates, their reliable determination at low concentration levels is difficult. In this study, a xanthate pretreatment method was optimized and applied for the determination of ethyl xanthate (EX^−^) by high performance liquid chromatography–inductively coupled plasma tandem mass spectrometry (HPLC–ICP-MS/MS). Ethyl xanthate was oxidized to diethyl dixanthogen ((EX)_2_) by triiodide (I_3_^−^) in aqueous solution and the formed (EX)_2_ was extracted into n-hexane. Important experimental parameters, including pH, I_3_^−^ amount, and oxidation time, were optimized and the detection limit of 0.29 mg L^−1^ for potassium ethyl xanthate was obtained. During the optimization experiments, it was found that the oxidation reaction resulted in multiple products, decreasing the efficiency of (EX)_2_ formation and, therefore, the sensitivity of the method. The proposed method was applied to wastewater samples with recoveries of 105–106%. This study provides a selective method for the determination of ethyl xanthate and introduces novel information on the parameters affecting the oxidation of xanthates.

**Graphical abstract:**

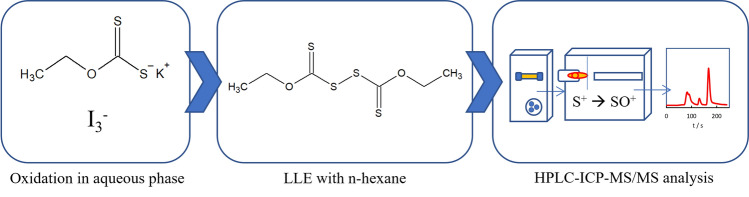

## Introduction

Xanthates are *O*-esters of dithiocarbonate that play a fundamental role in the processing of sulfide minerals. They are used as collectors in flotation processes where their function is to create a hydrophobic layer around the mineral particle promoting the particle’s adhesion to air bubbles [1]. Xanthates are also used as precursors in a variety of different chemical syntheses [2–4]. However, xanthates and especially their decomposition product, carbon disulfide (CS_2_), are hazardous to the environment. Xanthates are also known to react with heavy metals to create complexes that can enhance their bioaccumulation [5, 6]. Xanthates are listed as contaminants of emerging concern (CEC) in mine water even though their toxicity has already been known for decades. New concern over xanthates has risen particularly regarding their possibly persistent aquatic toxicity in cold climates [6, 7]. There have also been changes in the discharge limits for CS_2_, for example, in the US and Canada, where the new recommended limit is 1 mg L^−1^ [8].

Xanthates are weak conjugate bases of xanthic acids. They are known to be unstable in aqueous solutions, and they are typically decomposed through the following reaction mechanisms (Eqs. 1–3) that are widely accepted [9, 10]: In neutral and acidic conditions xanthate decompose fast through Eqs. 1 and 2, where xanthate first protolyzes forming xanthic acid, which then decomposes to alcohol and CS_2_. In basic conditions (Eq. 3), the decomposition rate decreases and other products such as carbonates are formed.

1 $${\mathrm{ROCS}}_{2}^{-}+{\mathrm{H}}_{2}\mathrm{O}\to {\mathrm{OH}}^{-}+{\mathrm{ROCS}}_{2}\mathrm{H},$$

2 $${\mathrm{ROCS}}_{2}\mathrm{H}\to \mathrm{ROH}+{\mathrm{CS}}_{2},$$

3 $$6\mathrm{ ROC}{\mathrm{S}}_{2}^{-}+3 {\mathrm{H}}_{2}\mathrm{O}\to 6\mathrm{ ROH}+{\mathrm{CO}}_{3}^{2-}+3\mathrm{ C}{\mathrm{S}}_{2}+2 {\mathrm{CS}}_{3}^{2-}.$$

Recent studies related to the reactions of xanthates have generally focused on these decomposition reactions and how to efficiently remove xanthate residues from the tailings. Hydrogen peroxide has been shown to decompose xanthate into carbonates and sulfates that have less environmental impacts than other xanthate derivatives [11]. This approach has been recently applied by García-leiva et al., where they used Fenton processes to decompose ethyl xanthate [12]. Ozone combined with UV radiation has also been studied for xanthate degradation purposes [13].

In addition to the products formed in the decomposition reactions, xanthates are susceptible to oxidation processes where dixanthogens, perxanthates, and monothiocarbonates are also formed. Dixanthogens are dimers formed from two xanthate anions, and they are readily formed on the surfaces of some minerals, such as pyrite and arsenopyrite, during the flotation process. It has been studied that on the surface of oxidized pyrite, the adsorption of xanthate occurs through oxidation of xanthate ions to dixanthogen [14]. This oxidation is coupled with the reduction of Fe(III) species released from the mineral surface [15]. Additionally, sulfoxy species such as thiosulfates and tetrathionates present in the flotation solutions can affect the formation of dixanthogens [16].

Dixanthogen can also be formed using additional oxidants. Triiodide oxidation has been used more frequently, even though ammonium persulfate has also been used for the preparation of dixanthogen [17, 18]. The kinetics and mechanism of xanthate oxidation to dixanthogen with triiodide has been studied by Cox and Natarajan [19]. They propose that the oxidation occurs through an intermediate product shown in Eqs. 4 and 5. The first step where the intermediate xanthate-iodine product forms is fast, and the reaction rate is determined by the second step where dixanthogen is formed.

4 $${\mathrm{ROCS}}_{2}^{-}+{\mathrm{I}}_{2}\rightleftharpoons {\mathrm{ROCS}}_{2}\mathrm{I}+{\mathrm{I}}^{-},$$

5 $${\mathrm{ROCS}}_{2}\mathrm{I}+{\mathrm{ROCS}}_{2}^{-}\to {\left({\mathrm{ROCS}}_{2}\right)}_{2}+{\mathrm{I}}^{-}.$$

Even though xanthates have been used in the mineral processing from the early twentieth century, there are still gaps in the knowledge even regarding simple decomposition behavior of xanthates [9, 20]. This is partly due to difficulties in their reliable quantitation. These challenges derive from the unstable nature of xanthates. Traditional analysis methods, such as spectrophotometric and titrimetric methods, which are used for monitoring xanthate concentration levels during the flotation process are typically non-selective and their detection limits are in the mg L^−1^ range [21]. New analysis methods are needed for the determination of xanthates from, for example, environmental water samples where the concentrations are very low [6, 7, 22]. Additionally, more selective methods are needed to study the reactions of xanthates reliably. Previously, selective methods have typically been based on liquid chromatography and they have been developed in the 1990s or late 1980s [23–27]. Lately, only few improved methods such as capillary electrophoresis (CE) have been proposed for the selective determination of xanthates [28].

In this study, the oxidation reaction of ethyl xanthate to diethyl dixanthogen was studied. The method is based on the studies of Zhou et al. [25], and the original method was applied in our recent study to investigate if high performance liquid chromatography–inductively coupled plasma tandem mass spectrometry (HPLC–ICP-MS/MS) could be used for the determination of xanthates [29]. One advantage of the developed method is its element-selective detection, which can give novel information on the reactions of xanthates. However, it was discovered that the sample pretreatment method needed further optimization to achieve accurate results. The main goal of this study was to explore how different experimental variables affect the oxidation process. The experimental conditions, including sample pH, oxidation time, and triiodide amount, were optimized to obtain linear response and maximize the yield of diethyl dixanthogen. Matrix effects were also studied, and, finally, the optimized process was applied as a sample pretreatment method for quantitation of xanthates by HPLC–ICP-MS/MS from wastewater samples.

## Experimental

### Materials and reagents

Ethyl xanthate solutions were prepared from potassium ethyl xanthate (KEX, ≥ 97.0%, Alfa Aesar, Kandel, Germany). Diethyl dixanthogen ((EX)_2_, ≥ 98.0%, MedChemExpress, NJ, USA) was used as a reference material. Analytical grade iodine (I_2_) and potassium iodide (KI) were used for the preparation of triiodide solution. Analytical grade sodium hydroxide (NaOH), ammonium hydroxide (NH_4_OH), nitric acid (HNO_3_, 69%), potassium dihydrogen phosphate (KH_2_PO_4_), acetic acid (CH_3_COOH), sodium acetate (CH_3_COONa), boric acid (H_3_BO_3_), and potassium chloride (KCl) were used for pH adjustment and preparing buffer solutions. Sodium nitrate (NaNO_3_) was used for adjusting the ionic strength. The reagents used for triiodide oxidation and pH adjustment were purchased from Merck (Germany). Gradient or HPLC grade methanol and n-hexane (Honeywell, Seelze, Germany) were used as the mobile phase in the HPLC system and as the extraction solvent, respectively. All aqueous solutions were prepared with ultrapure water (18 MΩ cm) purified with Millipore Gradient system. Matrix effects were evaluated using analytical grade Ca^2+^, Cu^2+^, Fe^2+^, Zn^2+^, and SO_4_^2−^ stock solutions (1000 mg L^−1^, Merck, Germany) from which appropriate dilutions were made.

Wastewater sample collected from Oulu Mining School (OMS) Minipilot was used for testing the applicability of the method. The Minipilot is based on the Pyhäsalmi mine concentrator [30]. The sample was collected from a tailings tank in which the water remained from a flotation process was stored. The sample was stored in a refrigerator (6 °C) and its pH was measured to be 6.7.

### Analytical method

**Fig. 1:**
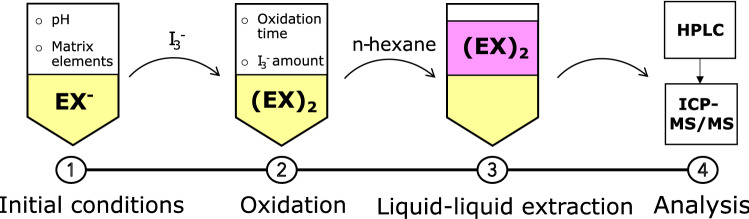
Schematic representation of the phases included in the developed method and studied variables in sample pretreatment for the determination of ethyl xanthate (EX^−^) as diethyl dixanthogen ((EX)_2_) by HPLC–ICP-MS/MS

The determination of ethyl xanthate consists of four phases presented in Fig. 1: Initial conditions in aqueous sample solution, oxidation, liquid–liquid extraction (LLE), and analysis. The first three steps were the focus of this study, and the HPLC–ICP-MS/MS-based analysis method was developed and optimized in a previous study [29]. The experimental parameters studied in each step are also presented in Fig. 1.

In the first step, solid KEX was dissolved in ultrapure water. A stock solution of 1 g L^−1^ KEX was prepared from the solid reagent from where the necessary dilutions were made. Fresh KEX working solutions were prepared daily and the stock solution was used for a maximum of two days. The initial pH of the KEX solutions was around 6–8 depending on the concentration. Higher concentration resulted in higher pH value. The pH of the solution was adjusted using sodium hydroxide (NaOH, to pH 12) or buffer solutions: acetate buffer (CH_3_COOH/CH_3_COO^−^) for pH 4, phosphate buffer (H_2_PO_4_^−^/HPO_4_^2−^) for pH 7, and borate buffer (H_3_BO_3_/H_2_BO_3_^−^) for pH 10. The pH adjustment was done prior to the addition of KEX to minimize the decomposition of KEX.

Next, triiodide solution (0.010 M I_2_, 0.20 M KI) was added to the sample. The amount of triiodide solution was either fixed to 50–500 μL per 3 mL of sample corresponding to 11–110 μmol of total iodine, or it was added gradually until the yellow color persisted in the sample. Oxidation time at room temperature ranged from 0 to 24 h. In optimized conditions, the sample pH was adjusted to 7; 200 μL (44 μmol) of I_3_^−^ was added to 3 mL sample, and the sample was oxidized for 1 h. After oxidation, LLE was done using n-hexane. LLE was typically done in 1:1 volume ratio. Formed (EX)_2_ and other non-polar species were extracted, and polar species remained in the aqueous solution. An aliquot was taken from the pretreated sample, and it was analyzed using HPLC–ICP-MS/MS. Twofold and fourfold preconcentration were also applied by decreasing the volume of *n*-hexane: 0.8 or 1.6 mL of *n*-hexane was added to 3.2 mL of aqueous sample.

Matrix effects of different ions to the sample pretreatment process were studied in aqueous solutions without pH adjustment. The matrix effects were studied for 10 mg L^−1^ KEX solution, and the concentration ranges of the studied species were as follows: Ca: 100–550 mg L^−1^, Cu: 10–100 mg L^−1^, Fe: 5–100 mg L^−1^, Zn: 0.5–100 mg L^−1^, and sulfate: 100–1000 mg L^−1^. Direct measurement of KEX by UV–Vis spectrophotometry was used as a comparative method. Standards and samples for direct measurement were prepared in 1% (v/v) NH_4_OH solution to decrease the decomposition of KEX [31, 32].

### Instrumentation

The 1260 Infinity II HPLC system (Agilent Technologies, Santa Clara, CA, USA) used for reversed-phase separation consisted of an 1100 Series Autosampler, a Bioinert 1260 II Pump, and a Multicolumn Thermostat. The system was equipped with a Poroshell 120 EC-C18 (2.1 × 50 mm, 2.7 µm) column. A Variable Wavelength Detector (VWD) was used for spectrophotometric detection of (EX)_2_ at 240 nm.

The HPLC system was primarily coupled with 8900 Triple Quadrupole ICP-MS (Agilent Technologies) instrument. The ICP-MS was used to detect (EX)_2_ and the other reaction products based on their sulfur content. The ICP-MS was operated in MS/MS mode using O_2_ (≥ 99.999%) as reaction gas. This allows the detection of ^32^S^+^ as ^32^S^16^O^+^ which resolves the spectral interferences due to overlap of ^16^O_2_^+^ with ^32^S^+^. ^127^I^+^ was measured in the same run. The instrumental method was optimized and evaluated for the determination of sulfur in a previous study for both HPLC–UV and HPLC–ICP-MS/MS [29]. The key operating conditions are presented in Table 1. The Shimadzu UV-1800 spectrophotometer was used for cross-checking the developed method. The absorption was measured at 301 nm using a 1 cm quartz cell.

**Table 1:** Operating conditions of 1260 Infinity II HPLC and 8900 Triple Quadrupole ICP-MS systems

Instrument	Parameter	Value
HPLC	Mobile phase composition	80:20 (v/v) Methanol–Ultrapure water
	Mobile phase flow rate	0.4 mL min^−1^
	Column temperature	20 °C
	Injection volume	6 μL
VWD	Wavelength	240 nm
ICP-MS/MS	RF power	1550 W
	Gas flow rate	
	Nebulizer gas	0.52 mL min^−1^
	O_2_ option gas	20% (~ 0.2 L min^−1^)
	O_2_ reaction gas	15% (~ 0.225 mL min^−1^)
	Sampling depth	10 mm
	Data acquisition mode	Time resolved analysis
	Total acquisition time	4 min
	Monitored masses	^32^S^+^ → ^32^S^16^O^+^ (mass-shift) ^127^I^+^ (on-mass)

### Data analysis

The limit of detection (LOD) and the limit of quantitation (LOQ) were determined using the standard deviation of the linear calibration curve intercept. The limit of detection was calculated from the following equation: $\mathrm{LOD}=3{\sigma }_{\mathrm{intercept}}/S$, where *S* is the slope of the calibration curve. *σ*_intercept_ was calculated as ${\sigma }_{\mathrm{intercept}}={s}_{y/x}\surd ((\sum {x}_{i}^{2} )/(n\sum {\left({x}_{i}-\overline{x } \right)}^{2}))$, where *s*_*y*/*x*_ is the residual standard deviation [33]. The limit of quantitation was calculated similarly to LOD, using 10 as the multiplier.

The efficiency of the sample pretreatment was evaluated using a linear standard curve of commercial (EX)_2_ as the reference. The (EX)_2_ concentrations were measured from pretreated KEX samples with initial KEX concentrations of 1, 3, and 5 mg L^−1^. Three replicate pretreatments were made at each concentration level. The average value of the measured concentration was compared to the theoretical maximum value of (EX)_2_ using the following equation: ${R}_{\mathrm{pretreatment}}=({C}_{\mathrm{measured}}/{C}_{\mathrm{theoretical}})\bullet 100\%$.

The recovery of the spiking experiments of real samples was calculated using the following equation: ${R}_{\mathrm{spiked}}=(({C}_{\mathrm{spiked}}-{C}_{\mathrm{initial}})/{C}_{\mathrm{calculated}})\bullet 100\%$, where *C*_spiked_ and *C*_initial_ are the measured (EX)_2_ concentrations in the sample after and before spiking, respectively. *C*_calculated_ is the theoretical maximum concentration for (EX)_2_ formed in a stoichiometric oxidation reaction of KEX.

A two-sided *t* test (*P* = 0.05) for comparison of two experimental means was used to compare results from HPLC–ICP-MS/MS and UV–Vis spectrophotometric measurements [33].

## Results and discussion

### Speciation by HPLC–ICP-MS/MS and –UV

**Fig. 2:**
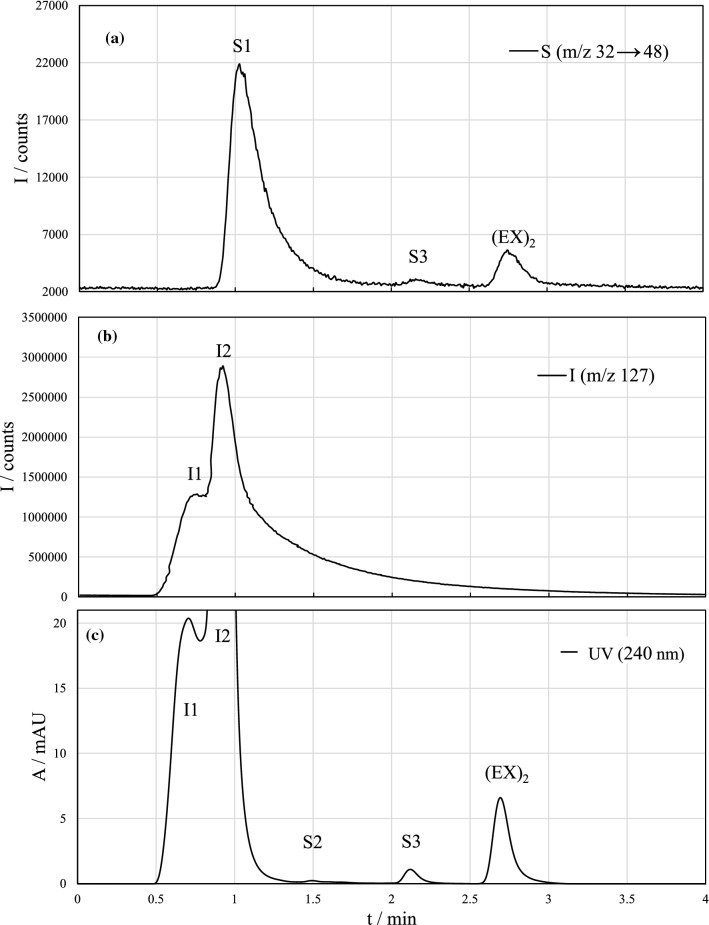
Chromatograms for oxidized KEX solution (5 mg L^−1^) by HPLC–ICP-MS/MS at **a** *m/z* 32 → 48 (S^+^ → SO^+^), **b** *m/z* 128 (I^+^), and **c** HPLC–UV (*λ* = 240 nm). pH of the sample was around 7; no buffer was added. Peaks I1 and I2 originate from triiodide and peaks S1–S3 from ethyl xanthate. (EX)_2_ = diethyl dixanthogen

The aim of the sample pretreatment process was to convert EX^−^ to (EX)_2_ by oxidation with triiodide. Typical chromatograms by HPLC separation and ICP-MS/MS and UV-detection are shown in Fig. 2. In total, six recurring peaks were detected. (EX)_2_ is the desired reaction product and it can be detected with both detection methods at retention time of 2.7–2.8 min. This has been confirmed using commercial (EX)_2_ as a reference material, which elutes at the same time [29]. The UV-spectra of the (EX)_2_ peak has also been measured and it compares well with previous literature giving absorption maxima at 238 and 286 nm [17, 34, 35].

The unknown compounds were named as I1–I2 and S1–S3 based on their detection by ICP-MS/MS either as iodine or as sulfur. Likely, the same compounds are present in the chromatograms obtained by UV-detection; so, the same nomenclature was used. Peaks S1–S3 originate from EX^−^, and their peak area ratio was proportional to (EX)_2_, depending on the experimental conditions of the oxidation process such as oxidation time and pH. UV-spectra were also measured for compounds S2 and S3: S2 had a sharp maximum at 272 nm, and S3 yielded similar spectrum as (EX)_2_, with absorption maximum values at 238 and 284 nm. Peaks I1 and I2 originated from the oxidant and can be detected both by UV absorption at 240 nm and as iodine at *m/z* 127 by ICP-MS/MS. These peaks were also detected from blank samples, and the response of signal I2 decreased with increasing EX^−^ concentration when the same amount of triiodide solution was used. This indicates that the signal is due to elemental iodine (I_2_) that is reduced to I^−^ during xanthate oxidation, and I^−^ is not extracted to the organic phase. Peak I1 eluted nearly in void volume, and so it could be a combination of small, possibly ionized iodine species.

The EX^−^ based compounds found in the extracted samples should be non-polar, which narrows down the likely compounds. No unoxidized EX^−^ was found in the extracted samples, and it is unlikely that ionic decomposition products of xanthates, such as thiosulfate, monothiocarbonate, or perxanthate, would be extracted and detected from the organic phase.

Formation of S1 and S2 depends on the sample pH, which is discussed in more detail in “Effect of pH”. The measured absorption maximum of S2 (272 nm) indicates that it could be ethyl xanthic acid [36]. The identity of S1 is difficult to confirm with the obtained information. However, it was hypothesized that it could be CS_2_. S1 was not detected by UV-detector, although CS_2_ is UV-active having absorbance maximum at 206 nm [37]. This could be partly due to overlap with I2 in UV-detection: The retention time of I2 is approximately 0.90 min, and for S1 it is 1.03 min. CS_2_ is also easily vaporized, which results in enhanced sample introduction efficiency to ICP-MS with solution nebulization when compared, for example, to (EX)_2_. This could explain the high signal intensity by ICP-MS/MS detection. The identity of signal S3 was also not confirmed. Its relatively high retention time and similar absorption spectra to (EX)_2_ indicates similar structure to (EX)_2_. More elaborate studies would be necessary to confirm the previous hypotheses.

Some peak tailing was present in the method, especially for the early-eluting compounds. No pH buffers or other modifiers were used in the mobile phase, which possibly increases tailing for part of the compounds. (EX)_2_ being a neutral molecule, pH of the mobile phase should not have a significant effect on peak tailing. The tailing of the (EX)_2_ peak might be partly related to the sample solvent considering the used solvent (hexane) is stronger than the mobile phase. Also other factors, such as extra-column volume from tubes and fittings, and column wearing out, may account for peak broadening [38].

**Fig. 3:**
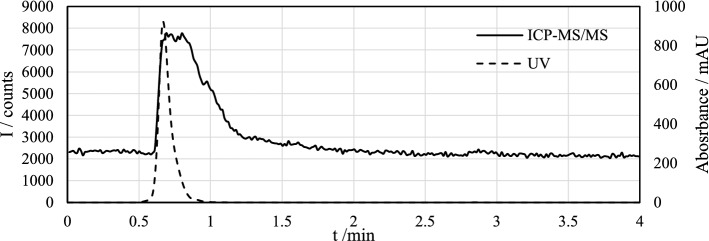
Chromatograms of aqueous phases after liquid–liquid extraction with n-hexane by HPLC–UV (*λ* = 240 nm) and -ICP-MS/MS (*m/z* 32 → 48)

Aqueous phases were also analyzed after the extraction and the chromatograms are presented in Fig. 3. No significant amount of (EX)_2_ was found in the aqueous phase indicating good extraction efficiency. Early-eluting peaks detected by ICP-MS/MS likely consisted of thiocarbonates or unoxidized EX^−^ that could not be separated with this method. The high UV response could be additionally explained by iodide eluting at the same time as the remaining xanthate species.

### Effect of pH

**Fig. 4:**
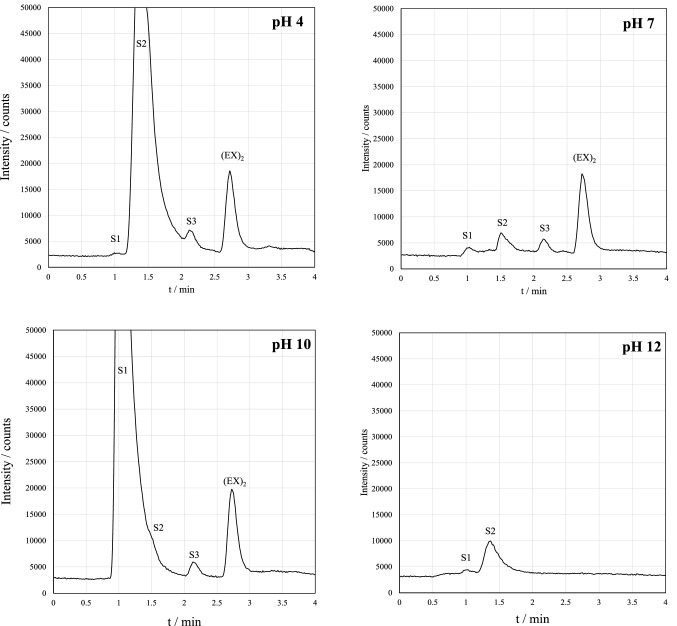
Chromatograms of pretreated KEX samples (10 mg L^−1^) at different aqueous phase pH values. Chromatograms were obtained with HPLC–ICP-MS/MS (*m/z* 32 → 48)

In this work, the oxidation was done in aqueous solution. Previously, triiodide oxidation had been applied in methanol–water solution [24, 25]. To simplify the pretreatment process and make it better applicable for real samples, the sample dilution with methanol was excluded. The sample pH was studied to see its effect on the response of (EX)_2_, and typical chromatograms at different pH levels for 10 mg L^−1^ KEX samples are shown in Fig. 4. The main observation was that the (EX)_2_ response did not change significantly between pH values between 4 and 10. However, when using pH 12, no (EX)_2_ was detected at the studied concentration level; pH 12 was then tested with higher initial KEX concentration (50 mg L^−1^) in which (EX)_2_ was detected but with decreased response compared to other pH values. This indicates that the formation of (EX)_2_ is not completely prevented in highly alkaline solutions even though the effect is significant.

In addition to (EX)_2_, varying responses of other compounds were found at different pH values: pH 4 resulted in high response of S2, whereas the response of S1 was high at pH 10. However, results in neutral pH show the highest response of (EX)_2_ when compared with the other products. The absolute response of (EX)_2_ was still very similar in all samples.

In practice, it would be unnecessary to adjust the pH below 7 because it does not increase (EX)_2_ response. Xanthates also decompose rapidly in acidic solutions, making the pretreatment process less robust. These results suggest that pH values between 7 and 10 are suitable for the pretreatment process, and the pH adjustment does not need to be extremely precise. pH 7 was chosen for the later experiments because it was found to result in consistently similar results regarding all detected peaks. At pH 4 and 10, the response of S1 and S2 was found to vary inconsistently, but the reason for this phenomenon was not studied further.

### Effect of oxidation time

**Fig. 5:**
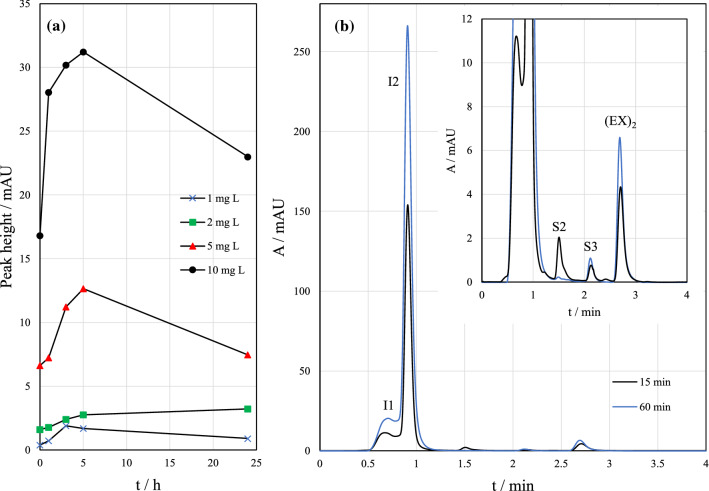
**a** Effect of oxidation time on the response of diethyl dixanthogen ((EX)_2_) at different potassium ethyl xanthate (KEX) concentrations. **b** Chromatograms of 5 mg L^−1^ KEX sample after 15- and 60-min oxidation time, no pH adjustment. Measured by HPLC–UV (*λ* = 240 nm)

The effect of oxidation time on the response of (EX)_2_ was studied at concentration levels 1–10 mg L^−1^ by HPLC–UV (Fig. 5a). The response of (EX)_2_ was rapidly increased in 10 mg L^−1^ sample, if oxidation time was increased from 0 to 1 h, where 0 h implies that extraction was done immediately after the oxidant was added, sample was stirred, and the yellow color persisted (100 µL I_3_^−^/3 mL). Within 1–5 h, the increase of the response became less steep. The trend in response was slightly different with 5 mg L^−1^ KEX, where the fast increase was found between 1 and 3 h. The (EX)_2_ response did not stabilize but began to decrease probably due to decomposition and was significantly decreased after 24 h of oxidation. A 1-h oxidation time was selected for the following experiments because a high enough and stable response with a reasonable pretreatment time was obtained.

The oxidation time also affected the other reaction products. As shown in Fig. 5b, the intensities of all the other peaks except S2 increased with increasing oxidation time. Peak S1 was not detected with the UV-detector (see Fig. 2); so, its behavior is unknown. The decrease rate of S2 follows a similar trend as the increase rate of (EX)_2_ during the first few hours of oxidation. When the sample was oxidized for a longer period (up to 24 h), S2 response stayed constant.

### Effect of triiodide addition

In previous studies, the volume of triiodide solution was chosen by visual observation: The excess of oxidant was reached when the yellow color persisted in the solution [25]. In this study, the effect of triiodide addition was studied further. During the preliminary experiments, it was found that the response (peak area) of (EX)_2_ followed a quadratic trend at the studied concentration range (1–15 mg L^−1^) when the original method was applied, regardless of the sample matrix. Linear response was not achieved even when the studied concentration range was narrowed.

In the preliminary experiments, 20–90 μL of I_3_^−^ solution was added to 3 mL sample, depending on the KEX concentration, and the oxidation time was 10 min. This was modified by changing the added I_3_^−^ amount to 100 and 200 μL, while oxidation time was not changed. These experiments showed that the linearity was better with increased I_3_^−^ volume. Therefore, more elaborate tests on I_3_^−^ addition were made. The oxidation time in these experiments was chosen to be 1 h, because, as seen in Fig. 5, the oxidation reaction is also time dependent.

**Fig. 6:**
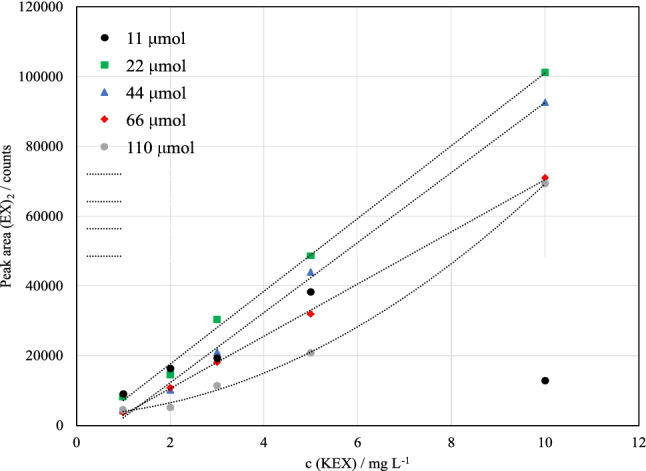
Response of diethyl dixanthogen ((EX)_2_) after oxidation process of potassium ethyl xanthate (KEX) when using different amounts of triiodide as the oxidant. Sample volume was 3 mL, no pH adjustment (natural pH 6*–*8). Measured by HPLC–ICP-MS/MS (*m/z* 32 → 48)

The effect of triiodide solution volume on the response of (EX)_2_ was studied over the range of 50–500 μL in 3 mL of sample with no pH adjustment when KEX concentration was 1–10 mg L^−1^. The triiodide solution volume corresponds to 11–110 μmol of total iodine added to the sample. As shown in Fig. 6 and Table 2, linear response (*R*^2^ > 0.99) was achieved with 22–66 μmol of iodine. The response of (EX)_2_ was also increased with the decreasing amount of triiodide. When 110 μmol of triiodide was used, the response again became nonlinear. When 11 μmol was added, the response of 10 mg L^−1^ sample was very low. This was probably due to the excess of EX^−^ in the solution instead of the excess of I_3_^−^, which affected the reaction equilibrium significantly.

**Table 2:** Equations for the standard curves of diethyl dixanthogen ((EX)_2_) with different triiodide additions by HPLC–ICP-MS/MS

Amount of I_3_^−^/μmol	Equation	*R*^2^
11	–	–
22	*y* = 10436*x* − 3296.5	0.9972
44	*y* = 10042*x* − 7815.4	0.9975
66	*y* = 7478.1*x* − 4324.5	0.9992
110	*y* = 598.78*x*^2^ + 657.98*x* + 2790.5	0.9987

*y* denotes peak area of (EX)_2_ and *x* the initial concentration of potassium ethyl xanthate (KEX)

It seems that adequate linearity and sensitivity can be achieved using 22–66 μmol of triiodide when the KEX concentration range is 1–10 mg L^−1^, and the oxidation time is 1 h. If I_3_^−^ is in too high excess in the solution, first the sensitivity and then the linearity begins to suffer. There could also be some interaction effects related to pH, oxidation time, and I_3_^−^ amount. Even though linear response was achieved with 22 μmol addition in samples with no pH adjustment, 44 μmol was necessary for samples buffered to pH 7.

### Analytical characteristics

The developed HPLC–ICP-MS/MS method was applied at a concentration range of 1–7 mg L^−1^ KEX, where the fitted linear equation *y* = 11690*x* − 1921 (*R*^2^ = 0.998) was obtained with 5 calibration points and each point was measured 3 times. In the equation, *y* and *x* denote the peak area of (EX)_2_ and concentration of KEX, respectively. The method LOD was 0.29 mg L^−1^, which was calculated using the presented linear calibration curve. This corresponds to 1.72 ng of KEX in 6 μL of the sample (injected to the HPLC system). The limit of quantitation was 0.96 mg L^−1^. The obtained LOD and LOQ were very reasonable when comparing them to the visual evaluation of the chromatographic peaks. Has the LOD been calculated using the standard deviation of the blank sample’s peak area, the resulting LOD would have been 0.054 mg L^−1^. Precision of the measurement ranged from 0.6% (3 mg L^−1^) to 9.8% (1 mg L^−1^), when *n* = 3.

The efficiency of the sample pretreatment process was evaluated by measuring (EX)_2_ concentrations from samples with known KEX additions against a standard curve of (EX)_2_. As shown in Table 3, 29–37% recovery of (EX)_2_ was achieved with the optimized conditions. This suggests that the oxidation efficiency is not very high, which could be due to various reasons. However, the efficiency is high enough to achieve low enough LOD and the pretreatment procedure is reproducible. The lack of efficiency is mostly related to the oxidation reaction because no significant dixanthogen losses were observed in the LLE step. The commercial (EX)_2_ was measured from methanol and samples from n-hexane, which could add some uncertainty to the results. However, its effect was estimated to be negligible compared to that of oxidation efficiency.

**Table 3:** Recovery of potassium ethyl xanthate (KEX) after oxidation as diethyl dixanthogen (EX)_2_

Initial KEX concentration (mg L^−1^)	Calculated (EX)_2_ (mg L^−1^)	Measured (EX)_2_ ± SD (mg L^−1^)	Recovery ± SD (%)
1.0	0.76	0.22 ± 0.01	29 ± 2.0
3.0	2.27	0.85 ± 0.03	37 ± 1.3
5.0	3.78	1.40 ± 0.13	37 ± 3.5

(EX)_2_ concentration was measured by HPLC–ICP-MS/MS using commercial (EX)_2_ for calibration (3 replicate pretreatments)

### Matrix effects

Regarding the sample matrix, pH was observed to have a significant effect on the formation of (EX)_2_. The effect of other possible matrix species was studied one by one to identify the important species. The studied matrix species were SO_4_^2−^, Ca^2+^, Fe^2+^, Zn^2+^, and Cu^2+^. No other species except Zn^2+^ had any effect on the formation of (EX)_2_. The response of (EX)_2_ disappeared almost completely (> 99%) at all studied Zn concentration levels (0.5–100 mg L^−1^). The response of other xanthate derived species (S1–S3) was also decreased significantly.

Transition metals are known to affect the decomposition of xanthates to CS_2_. For example, Fe^3+^ has been found to promote decomposition whereas Cu^2+^ and Zn^2+^ suppresses it. The suppression happens due to the formation of relatively stable metal-xanthate complexes [39, 40]. It is possible that in these conditions ethyl xanthate formed a complex with Zn^2+^ and oxidation to diethyl dixanthogen did not occur. However, it is interesting that the same phenomenon was not observed with Cu^2+^.

### Application

The applicability of the method to real samples was assessed by spiking KEX to a wastewater sample collected from the tailings tank of OMS flotation process. The elemental composition of the sample was measured using ICP-OES and the main elements found were sulfur (510 mg L^−1^) and calcium (590 mg L^−1^). Additionally, 0.4 mg L^−1^ of zinc was detected. Iron and copper were also measured, but their concentrations were below the detection limit of the method. The KEX concentration in the sample was also below the LOD before the spiking experiments.

The results of the spiking experiments are shown in Table 4: Good recoveries were achieved with both 1 and 3 mg L^−1^ additions, even though 1 mg L^−1^ is near the method LOQ. Although some zinc was detected from the wastewater sample, the formation of (EX)_2_ was not suppressed as was observed for pure Zn^2+^ solution (“Matrix effects”). This could be due to the different speciation of zinc in the studied wastewater sample. This emphasizes the importance of matrix effect studies when applying this method to different sample types such as environmental samples. For example, Vega and Weng studied that in river water 52% of the total zinc existed as free Zn^2+^ [41].

**Table 4:** Recoveries of KEX from flotation wastewater sample by HPLC–ICP-MS/MS using 1–4 times preconcentration in the extraction step (*n* = 3)

Spiked KEX concentration (mg L^−1^)	Preconcentration factor	Measured KEX concentration ± SD (mg L^−1^)	Recovery ± SD (%)
1.0	1	1.07 ± 0.010	106 ± 10
	2	2.19 ± 0.175	110 ± 9
	4	4.83 ± 0.140	121 ± 3
3.0	1	3.24 ± 0.157	105 ± 5
	2	6.25^a^	104^a^

^a^*n* = 1

Preconcentration could be done in the LLE step to improve the detection limit of the method. This was preliminarily tested with the wastewater samples, and good results were obtained with twofold preconcentration. Fourfold preconcentration was tested for 1 mg L^−1^ spike, which resulted in slightly too high recovery. However, these results indicate that preconcentration can be applied to real samples if lower detection limits are needed.

To study the specificity of the developed method, the wastewater sample spiked with 3 mg L^−1^ was also analyzed by UV–Vis spectrophotometer. According to the *t* test with unequal variances, KEX results obtained for spiked sample by HPLC–ICP-MS/MS (3.24 ± 0.157 mg L^−1^, *n* = 3) and UV–Vis spectrophotometric (3.13 ± 0.01 mg L^−1^, *n* = 3) methods do not differ significantly (*p* < 0.05).

## Conclusions

A sample pretreatment method for the determination of ethyl xanthate (EX^−^) was optimized in this study. Ethyl xanthate was oxidized in aqueous solution by triiodide to diethyl dixanthogen ((EX)_2_) and extracted to n-hexane prior to the determination by HPLC–ICP-MS/MS. From the studied parameters, it was found that pH, oxidation time, and the amount of triiodide had the most significant effects in the sample pretreatment process. In the optimized conditions, the pH of KEX samples (1–10 mg L^−1^) was adjusted to 7; 200 μL of triiodide was added to 3 mL of sample, and the sample was oxidized for 1 h.

In optimized conditions, the LOD for KEX was 0.29 mg L^−1^ when no preconcentration was applied. An amount of 1 and 3 mg L^−1^ of KEX were added to a wastewater sample, and their recoveries were excellent: 106% and 105%, respectively. In addition, preliminary tests showed that at least twofold preconcentration can also be done during the LLE step, which allows the determination of xanthates even at lower concentration levels.

While optimizing the pretreatment conditions, information about the oxidation reaction was also obtained. The efficiency of the sample pretreatment process to form dixanthogen was 29–37%. Formation of other reaction products was observed as additional peaks in the chromatographic analysis. The identity of these products was discussed based on the obtained results and previous literature. However, these side products were not reliably identified.

This pretreatment method combined with the determination by HPLC–ICP-MS/MS offers a sensitive and selective method for studying xanthates at low concentration levels, for example, from mining wastewaters and natural waters. The results also show that the oxidation reactions of xanthates are complex, resulting in multiple reaction products. More elaborate studies are needed to reliably identify all species formed in the oxidation reaction.

## Data Availability

The datasets generated during and/or analyzed during the current study are available from the corresponding author on reasonable request.
